# Developing a global core competency framework for clinical research

**DOI:** 10.1186/1745-6215-16-S2-P191

**Published:** 2015-11-16

**Authors:** Tamzin Furtado, Amélie Julé, Liam Boggs, Francois van Loggerenberg, Victoria Ewing, Trudie Lang

**Affiliations:** 1 The Global Health Network, Oxford, UK; 2 Centre for Tropical Medicine and Global Health, University of Oxford, Oxford, UK

## 

Lack of recognition for working in clinical research is widely cited as an impediment to its conduct. There is a lack of career structure for the many roles involved (investigators, trial managers, nurses, etc.), and a lack of understanding of who does what. Competency frameworks exist for some individual job roles, but these are infrequent; thus the need for a global framework describing roles and responsibilities in a research team. This would facilitate appraisal of staff, promote career development by highlighting acquired skills, and illuminate areas where training opportunities are lacking.

In this project, we combine 28 frameworks created by external groups, with information from 116 job descriptions obtained from partners in clinical trial units worldwide and from the web, to create a widely-encompassing framework derived from 11 different roles. Using qualitative analysis software, we systematically assess the activities performed by the clinical research team to categorise them and define underlying competencies - knowledge-, skill- or task-based. The resulting framework counts 50 competencies required throughout the trial lifecycle, from assessment of scientific literature to results dissemination via project management, public engagement or grant application. It is applicable to studies that may differ in design, geographical location, disease, etc., and can be adapted to the particular needs of specific projects or roles.

This “Global Core Competency Framework for Clinical Research” is currently being validated through consultations with experts in collaboration with WHO-TDR, in order to refine its completeness and accuracy, as well as its practicability before it is deployed into the field.

